# Provider costs of professional COVID-19 rapid antigen testing in low-income settings

**DOI:** 10.1371/journal.pgph.0005251

**Published:** 2025-10-08

**Authors:** Collin Mangenah, Lucky G. Ngwira, Obinna Ekwunife, Linda Sande, Gabrielle Bonnet, Progress Chiwawa, Linea Mashoko, Desiderata Nkhoma, Norah Mwase, Elvis Isere, Itai Kabonga, Constancia Watadzaushe, Rudo Chinoruma, Yasmin Dunkley, Augustine Choko, John S. Bimba, Brian Maponga, Noah Taruberekera, Euphemia Sibanda, Frances M. Cowan, Karin Hatzold, Elizabeth L. Corbett, Gabrielle Bonnet

**Affiliations:** 1 Centre for Sexual Health and HIV/AIDS Research, Harare, Zimbabwe; 2 Department of International Public Health, Liverpool School of Tropical Medicine, Liverpool, United Kingdom; 3 Malawi-Liverpool-Wellcome Trust Clinical Research Programme, Blantyre, Malawi; 4 Department of Clinical Pharmacy and Pharmacy Management, Nnamdi Azikiwe University, Awka, Nigeria; 5 Faculty of Infectious and Tropical Diseases, London School of Hygiene and Tropical Medicine, London, United Kingdom; 6 Population Solutions for Health, Harare, Zimbabwe; 7 Population Services International, Washington, District of Columbia, United States of America; PLOS: Public Library of Science, UNITED STATES OF AMERICA

## Abstract

World Health Organization recommends antigen rapid diagnostic tests (RDT) as point of care tests for severe acute respiratory syndrome coronavirus 2 (SARS-CoV-2) in suspected outbreaks when polymerase-chain-reaction testing is not accessible; to trace the extent of outbreaks; and in areas with widespread community transmission. Annual economic costs were estimated for professional SARS-CoV-2 testing as part of several COVID-19 testing use cases in Malawi, Nigeria and Zimbabwe. Symptom screening and antigen-based RDT was implemented as part of a multi-country, Unitaid/STAR 3ACP (Africa, Asia, America COVID-19 Prevention) funded project (April 2022-June 2023). Testing services were provided through trained health providers in outpatient departments of primary care facilities (Malawi and Nigeria) and two primary non governmental organisation (NGO) use cases separately targeting key population (KP) and the general population in Zimbabwe. Combined financial expenditure analysis and on-site micro-costing took the provider/health system perspective in 2025 US$. Per test average costs were $9.73 (range across sites: $5.49-$29.90) in Malawi, $13.99 ($11.64-US$18) in Nigeria and $10.11 ($4.19-$209.09) and $19.98 ($10.76-$56.40) in Zimbabwe for general population and key population clinics respectively. Average costs per positive case identified were $521 ($61-$800) in Malawi; $1,118 ($202.66-$4,804.45) in Nigeria; and $1,125 ($336-$ 1,762) and $187 ($161-$ 1,272) in Zimbabwe. Major cost contributors were test kits in Malawi, test kits and building (consultation room space costs) and storage in Nigeria and personnel and training in Zimbabwe. Excluding above site level costs, the average cost per SARS-CoV-2 test was $9.73 in Malawi, $13.99 in Nigeria and $10.70 and $9.79 in Zimbabwe. Integrating COVID-19 testing into existing sites can reach people at high risk of severe illness at a reasonable cost. For resource-limited settings where programmes are threatened by low fiscal space, costs might be reduced when scaling up, through greater spreading of startup and capital costs.

## Introduction

Coronavirus disease 2019 (COVID-19), a highly contagious and infectious disease caused by severe acute respiratory syndrome coronavirus 2 (SARS-CoV-2) was designated a global pandemic on 11 March 2020, following a swift global increase in the number of cases [1–3]. By September 2023, COVID-19 had resulted in substantial mortality and morbidity with 766 million confirmed cases and 7 million reported deaths globally [4–5]. In May 2023, the World Health Organization declared an end to COVID-19 as a public health threat following a 12-month downward trend in deaths due to the development of highly effective vaccines and oral antivirals and a substantial increase in population immunity to severe outcomes [5–7].

Together with the emergence of highly transmissible but less pathogenic Omicron strains, this led to testing programmes downscaling globally towards more targeted strategies aimed at infection-prevention in the highest risk settings and early diagnosis and antiviral administration for the highest risk individuals [6]. Antiviral treatment requires prompt diagnosis of infection in patients at the highest risk of hospitalization as oral antivirals are most effective if initiated within 5 days of symptom onset, before serious manifestations have developed [8].

Implementation of high volume testing strategies that are affordable and sustainable as part of rapid pandemic responses in low-income settings is in the interest of vulnerable populations, public health systems, societies and economies. By end 2021 however testing uptake lagged behind despite high virus circulation [6]. In the case of COVID-19 only 70 million tests had been reported by African countries (a small fraction of the continent’s 1.3 billion people) by October 2021, compared to 550 million tests in the US (total population 334.9 million in 2023) and over 280 million tests in the United Kingdom (total population 68.35 million in 2023). A study in South Africa showed that 749 of 1,200 tested individuals (62·4%) had at least one SARS-CoV-2 infection episode, and 87 of 749 (11·6%) were reinfected [9]. Lack of improvement over time suggested that most African countries had still not developed effective testing systems or strategies.

WHO first issued interim guidance supporting antigen-based rapid diagnostic test (Ag-RDT) use in September 2020, with guidelines updated in October 2021 to support greater reliance on professional Ag-RDTs without the need for confirmatory testing, and again in March 2022 to recommend self-testing strategies [10,11]. Ag-RDTs detect SARS-CoV-2 antigens in the upper respiratory tract in the first week of infection when the virus is actively replicating but symptoms are typically mild [12]. As such, positive Ag-RDTs indicate infectiousness as well as early infection likely responsive to oral antivirals if indicated, which may have important benefits for treatment [13]. It is also likely to provide a cheaper substitute to polymerase chain reaction testing to guide treatment of severe or critical patients [14].

A mixed-methods study evaluated feasibility, acceptability and cost of several use cases for COVID-19 antigen tests across three countries: Malawi, Nigeria and Zimbabwe. Cost is a key determinant of whether an intervention is scalable, particularly in low-income settings, and is the focus of this paper. Understanding economic costs and their drivers is particularly useful to help rationalise resource use and improve service provision efficiencies. We describe the economic costs of professional COVID-19 testing with SARS-CoV-2 Ag-RDTs across the three countries.

## Methods

### Intervention overview

SARS-CoV-2 Ag-RDT strategies including self-testing and linkage to treatment and prevention for symptomatic participants and asymptomatic people who were contacts of a known case were implemented across Malawi, Nigeria and Zimbabwe as part of a multi-country, Unitaid/STAR 3ACP funded project (April 2022-June 2023) [15]. Although self-testing was provided the costing reported in this paper focusses only on the provider delivered costing services.

### Setting characteristics

Key setting characteristics across the three countries, Malawi, Nigeria and Zimbabwe are presented in Table 1. While Malawi sites where all peri-urban, the Nigeria and Zimbabwe use case sites were exclusively urban.

**Table 1:** Key setting characteristics.

Country	Malawi	Nigeria	Zimbabwe
National population	17,563,749 [16]	223,800,000 [17]	15,178,957 [18]
COVID-19 confirmed cases [19]	88,986 (1%)	267,146 (<1%)	265,771 (2%)
Confirmed deaths [19]	2,686	3,155	5,718
Vaccine doses administered	8,359,717	130,894,625	13,935,112

## Overview of country specific COVID-19 Ag-RDT use cases

### Malawi

In Malawi, Population Services International trained healthcare workers to provide COVID-19 testing services at the outpatient departments of 12 peri-urban primary health clinic (PHC) facilities serving the general population. In Malawi both outpatient and inpatient services are provided as part of primary healthcare consisting of smaller-level facilities such as health centers and community and rural hospitals by midwife assistants, nursing assistants, and clinicians [20].

### Nigeria

In Nigeria, the Society for Family Health [32] worked on training primary care facility healthcare workers to implement routine diagnostic COVID-19 Ag-RDTs testing for symptomatic patients presenting to outpatient departments of primary health clinics (https://sfhnigeria.simplicant.com) [21].

### Zimbabwe

In Zimbabwe, screening for COVID-19 was conducted through two primary NGO use cases, key population and general population clinics.

#### General population clinics.

In the NGO run general population clinic use case, Population Solutions for Health (PSH) implemented provider-delivered COVID-19 testing integrated with their ongoing clinic and workplace-based programmes as part of their integrated New Start Centre network (https://psh.org.zw). PSH **i**ntegrated clinical services include HIV testing services, COVID-19 testing and vaccination, voluntary medical male circumcision [22], pre-exposure prophylaxis (PrEP), linkages to antiretroviral treatment (ART) or onsite same-day ART initiation for clients with limited access (e.g., KP sub-populations), tuberculosis screening, diagnosis treatment and isoniazid preventative therapy (IPT), cervical cancer screening and treatment, short-term and long-acting reversible contraceptives, sexually transmitted infection [23] screening and treatment and comprehensive services for survivors of violence.

#### Key population clinics.

Within the key population clinic use case run by Centre for Sexual Health and HIV/AIDS Research (CeSHHAR) Zimbabwe in conjunction with Ministry of Health and Child Care, sex workers presenting to 11 static clinics within Zimbabwe’s nationally scaled Key Populations (KPs) [24,25] programme for sex workers underwent screening for COVID-19 symptoms according to current standard of care. KPs who had symptoms or who reported being contacts of individuals who had tested positive for COVID-19 were offered provider-delivered testing using rapid antigen tests [26,27]. COVID-19 testing within KP clinics was offered alongside the following comprehensive services: condom and lubricant provision, syndromic STI screening and management, family planning, HIV testing, index and social/risk network testing for all consenting positives, ART, PrEP, adherence support and viral load monitoring (Table 2).

**Table 2:** Overview of use cases across the three countries.

Country	Malawi	Nigeria	Zimbabwe	
Use case/Model	Outpatients Department	Outpatients Department	NGO GP clinics	NGO KP clinics
Type of clinic	Primary health care	Primary health care	Standalone NGO	Standalone NGO
Tester*	Healthcare worker	Healthcare worker	Healthcare worker	Healthcare worker
COVID-19 testing programme start date	June 2022	June 2022	May 2022	April 2022
Location: rural (urban or peri-urban)	Peri-urban	Urban	Urban	Urban
Population served by clinic	General population	General population	Integrated SRH & HIV	Integrated SRH & HIV
Services offered	Integrated SRH & HIV	Integrated SRH & HIV	Integrated within ongoing programs and limited to people with COVID-19 symptoms	Symptomatic KP or contacts of confirmed cases presenting to the clinic
Target clients	Patients attending outpatient services in primary care facilities	Patients attending outpatient services in primary care facilities	Patients attending outpatient services in NGO KP clinic facilities	Patients attending outpatient services in NGO GP clinic facilities
Type of test*	Provider delivered rapid antigen testing	Provider delivered rapid antigen testing	Provider delivered rapid antigen testing	Provider delivered rapid antigen testing

NGO – Non governmental organisation; GP – General population; KP - Key population; HIV – human immunodeficiency virus; SRH – Sexual and reproductive health.

* Self-testing was also provided but those costs are not the focus of this paper.

## Economic evaluation

### Costing overview

We estimated the full economic cost (in 2025 US dollars) of providing COVID-19 testing from a health provider’s perspective, following international costing guidelines [28–31]. Costing took a comprehensive approach involving a site or facility level bottom-up micro-costing exercise (conducted during a month-long survey at each site) aimed at understanding COVID-19 testing service provision and quantifying each resource input consumed in the process, as well as site-level interviews. These allowed data collectors to identify predefined allocation-factors (testing staff based in each site, distance from project head office and monitoring and evaluation system data such as the proportion of provider COVID-19 tests conducted) for the top-down categorisation of implementation partner expenditures by input type and use case or model and testing site (S1 Table).

### Data collection process

At each facility, service utilization data for SARS-CoV-2 antigen rapid diagnostic testing were collected for a retrospective 12-month period (varying by country context) from programme records, monthly reports, registers maintained in each testing unit or service department. Healthcare providers were interviewed, and data was accessed for research purposes across all countries coterminously - between 15/04/2023 and 15/06/2023. To ensure that site records matched central databases on which cleaned and verified data was stored and to ensure data were de-duplicated, we worked closely with programme monitoring and evaluation teams. Trained health economics data collectors used a standardised costing tool to identify and quantify all resources required to perform a point of care antigen rapid SARS-CoV-2 diagnostic test. To ensure costing was comprehensive enough, it was preceded by observations and interviews with site managers to understand the nature of testing costs (direct medical and non-medical), the clinical screening pathway at each testing site, the type and number of health providers involved, quantity of test kits and other supplies used per test and annually. Cost data collection included start-up (financial records), capital (collected through an inventory survey conducted at screening points) and recurrent costs.

### Estimating start-up and capital costs

Startup costs included all costs incurred ahead of programme launch or first test conducted, while capital costs included all building space (both shared and directly used for testing), clinical tents for SARS-CoV-2 testing, medical and non-medical equipment, and furniture. Capital costs were classified as any input that can be used over time and whose useful life exceeds one year [31–33].

### Estimating recurrent costs

#### Personnel costs.

Personnel cost estimation was conducted using time and motion analysis (gold standard for measuring staff allocation of time through direct observation) to understand how much time health provider staff dedicated to SARS-CoV-2 antigen rapid diagnostic testing relative to other departments and the specific time taken on specific tasks involved in testing individual patients [34,35]. Upon arrival at each site data collectors requested permission from site managers to conduct time and motion observations on health providers during their month-long survey. However before conducting any time and motion observations health provider staff randomly selected from a staff roster or list of those providing testing services (we aimed for all or every second participant if more than six) were asked to provide written informed consent to be observed as they went about their work. Trained economics data collectors (1 per site) then observed health providers while they were conducting testing, recording how much time it took to conduct testing sessions.

As data collectors aimed to maintain confidentiality by staying outside consultation rooms, interviews with health provider staff were used to help breakdown the time it took to conduct specific testing tasks or activities within a testing session. Mean time estimates from this process were used not only to directly estimate provider time costs per test but also as an allocation factor for overhead personnel (supervision and other support personnel time) costs. Although data collectors did their best to assure staff of confidentiality it was deemed more appropriate to collect data on monthly salaries of each staff grade involved in screening from the Human Resources Departments rather than engaging in frequently sensitive conversations between staff and outsiders (data collectors). It was felt that this process would ensure both consistency and accuracy. We then estimated a ‘salary per working hour’, by dividing the monthly salary by (i) the number of working days excluding weekends, public holidays and leave days, (ii) a working day divided by the number of working hours per day excluding lunch hour and tea breaks.

#### Resource requirements (RDTs and other supplies) for screening/testing.

Resources consumed during screening/testing were recorded by data collectors during the time and motion observations described above, by asking staff about supply use at the same time as the use of their time during the testing process. *S*upplies recorded in addition to RDTs include personal protective equipment (PPE), laboratory kits and reagents, and other materials.

#### Overheads.

Recurrent overhead costs (both head office and site level) including building running costs (water, electricity, telephone/communication, maintenance), vehicle running costs (servicing, fuel and lubricants), equipment maintenance and stationery (including printing and binding), cleaning and hygiene materials were estimated using financial expenditures provided by the finance departments of the respective country implementors.

#### Cost data analysis.

The standard approach to estimating full economic costs combines site-level bottom-up costing and top-down financial expenditure analysis to avoid underestimating either overheads when relying on the former or site level economic costs when relying on the latter [36,37]. General ledger financial expenditures were therefore requested from implementing partners (IP) and analysed line by line working with programme teams to first disaggregate expenses by input type or category and then conduct stepwise allocation to their respective distribution use case/model and clinic site. This approach has been shown to ensure that any inefficiencies, downtime, and wastage are properly accounted for [36].

Start-up, initial training and all other capital (including furniture, medical and non-medical equipment, and building space) costs were annualized using appropriate useful lifespans and at a 3% discount rate [38–41]. Equipment included staff swivel and visitor chairs for patients found in consultation rooms, waiting area benches, tables and filing cabinets regardless of whether purchased by the programme or pre-existing or donated by other concurrent programmes. For the replacement cost of buildings, we multiplied the cost per square metre (based on real estate estimates) by the area used for testing – including a percentage of all shared spaces. For each health facility, total annualised costs were divided by the number of tests performed. Overheads and recurrent costs were directly allocated by dividing total costs by the total annual number of tests.

The economic cost data were classified into above or at site level and by input type: start-up or initial training (incurred prior to the conduct of the first COVID-19 tests), capital and recurrent [42–44]. Annual programme costs were estimated for provider delivered COVID-19 test implementation timelines that varied by country context. All costs were converted to 2023 US dollars using average annual exchange rates. For each country, total COVID programme costs, total COVID-19 tests conducted and average cost per COVID-19 test conducted were estimated at the use case and site levels. Economies of scale were explored using the range of average site level costs.

### Sensitivity analysis

We conducted deterministic sensitivity analysis (univariate and scenario analyses) to assess whether our cost findings remained robust when key input parameters were varied to reflect different programmatic scenarios. We also present our costs both including and excluding above site level costs in order to more precisely compare costs across country use cases in the absence of financial expenditure data for some country uses cases [31].

### Ethics approvals

This cross-country cost analysis was part of major trials conducted following permissions granted by the respective ministries of health of Malawi, Nigeria and Zimbabwe. Ethical approvals for the parent studies were granted by the Malawi College of Medicine Research Ethics Committee (P.05/22/3649), Ethics Research Committees of Bingham University of Health Sciences and the National Health Research Ethics Committee in Nigeria, Medical Research Council of Zimbabwe (A2872) and externally by WHO Ethics Review Committee (CERC.0163; CERC.0160), and London School of Hygiene and Tropical Medicine (26931). Written informed consent was obtained for healthcare provider interviews to collect time and cost data. All study participants across the three country protocols provided written informed cosent to have data from their medical records used in research. All data were fully anonymized before access.

## Results

### COVID-19 programme testing outcomes

During the period of costing, a total of 30,137 COVID-19 RDT tests were conducted across the three country contexts respectively (Table 3). The average number of tests conducted per site was 2,199 (range: 550–4,033) across 12 sites in Malawi; 240 (range: 71–300) across 4 sites in Nigeria; 419 (range: 35–777) and 63 (range: 2–161) across 5 and 11 sites in the Zimbabwe use cases respectively.

**Table 3:** COVID-19 testing data across sites.

Country	Use case	Total number of COVID-19 tests (cross site)	Average number of COVID-19 tests (cross site)	Minimum number of tests (cross site)	Maximum number of tests (cross sites)
**Malawi**	PHC	26,386	2,199	550	4,033
**Nigeria**	PHC	959	240	71	300
**Zimbabwe**	NGO GP clinics	2,094	419	35	777
**Zimbabwe**	NGO KP clinics	698	63	2	161

PHC – Primary health clinic; NGO – Non governmental organisation; GP – General population; KP - Key population.

### Total COVID-19 programme costs and cost composition

The total COVID-19 testing programme costs were $256,672 (cross site range; $6,425 to $36,810) in Malawi and $13,418 (cross site range; $1,269 to $5,337) in Nigeria. For the Zimbabwe KP and GP use cases total programme costs were $41,766 (cross site range; $1,974 to $15,064) and $7,054 (cross site range; $234 to $1,406) respectively (Table 4).

**Table 4:** Total programme cost summary by input category (in 2025 US$).

Country	Malawi		Nigeria		Zimbabwe			
Country use case: Total tests Duration in months: Range	OPD*: 26,386 16 months: March 2022 to June 2023		PHC*: 959 12 months: May 2022 to June 2023		NGO* GP*: 2,094 12 months: May 2022 to June 2023		NGO* KP*: 698 12 months: May 2022 to June 2023	
Cost categories $ (by input)	Cost US$	%	Cost US$	%	Cost US$	%	Cost US$	%
**Fixed costs**								
**Start-up**								
** *Training***	*$15,962.65*	*6.2%*	*$775.07*	*6%*	*$2,036.00*	*5%*	*$2,313.16*	*33%*
** *Start-up other***	*–*	*–*	*$2,119.42*	*16%*	*$60.00*	*<1%*	*–*	
**Total startup costs**	$15,962.65	6.2%	$2,894.49	22%	**$2,096.00**	5%	**$2,313.16**	33%
**Capital costs**								
** *Buildings and Storage***	*$11,412.86*	*4%*	*$3,733.38*	*28%*	*$40.88*	*<1%*	*$19*	*<1%*
** *Equipment***	*$17,241.49*	*7%*	*$598.70*	*4%*	*$168.78*	*<1%*	*$68*	*1%*
**Total capital costs**	**$28,654.35**	**11.2%**	**$4,332.08**	**32%**	**$209.66**	**1%**	**$86.63**	**1%**
**Variable costs**								
**Recurrent costs**								
** *Personnel - HQ***	*–*	*–*	*–*	*–*	*$7,808.00*	*19%*	*$1,461*	*21%*
** *Personnel - Direct***	*$12,833.31*	*5%*	*$709.09*	*5%*	*$15,982.72*	*38%*	*$1,736*	*25%*
** *Personnel - Other***	*–*	*–*	*–*	*–*	*$5,189.00*	*12%*	*–*	
Test kits*	*$197,897.00*	*77%*	*$3,887.40*	*29%*	*$4,188.00*	*10%*	*$1,202*	*17%*
** *Supplies - Office stationery***	*–*	*–*	*–*	*–*	*$317.00*	*1%*	*–*	*–*
** *Supplies - Communications***	*–*	*–*	*–*	*–*	*$1,193.00*	*3%*	*–*	*–*
** *Supplies - Other***	*–*	*–*	*$143.48*	*1%*	*$2,896.00*	*7%*	*$222*	*3%*
** *Vehicle operation & transport***	*–*	*–*	*–*	*–*	*$1,587.00*	*4%*	*$33*	*<1%*
** *Building operation/maintenance***	*–*	*–*	*$1,451.60*	*11%*	*$136.00*	*<1%*	*–*	*–*
** *Recurrent training***	*–*	*–*	*–*	*–*	*$73.00*	*<1%*	*–*	*–*
** *Waste management - Other***	*–*	*–*	*–*	*–*	*$90.00*	*<1%*	*–*	*–*
** *Other recurrent costs***	*$1,324.32*	*1%*	*–*	*–*	*–*	*–*	*–*	*–*
**Total recurrent costs**	**$212,054.63**	**83%**	**$6,191.57**	**46%**	**$39,459.72**	**94%**	**$4,654.42**	**66%**
**Total programme costs**	**$256,671.63**	**100%**	**$13,418.14**	**100%**	**$41,765.38**	**100%**	**$7,054.21**	**100%**
**Costing outcomes**								
**# COVID-19 tests (all)**	26,386		959		2,094		698	
**Cost per COVID-19 test (all)**	$9.73		$13.99		$19.95		$10.11	
**# COVID-19-positive tests**	493		12		67		39	
**Cost per COVID-19-positive test**	$520.63		$1,118.18		$623.36		$180.88	

*Totals have been rounded to the nearest US$.

*Test kit costs excluding shipping and local distribution were $2.

*OPD – Outpatients Department; PHC – Primary health clinic; NGO – Nongovernmental organisation; KP - Key population; GP – General population.

**Fig 1:**
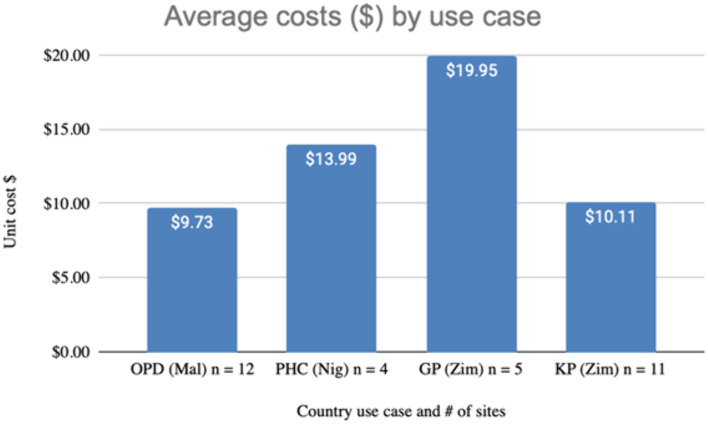
Average costs by use case.

Fig 1 shows the COVID-19 testing unit cost by each of the four types of testing country use cases side by side. The cost per COVID-19 test across the sites was $9.73 in Malawi, $13.99 in Nigeria and $10.11 and $19.95 in Zimbabwe respectively (Fig 1). Unit costs were lowest in the OPD use case in Malawi and highest in the integrated NGO GP use case in Zimbabwe.

### Key cost contributors

**Fig 2:**
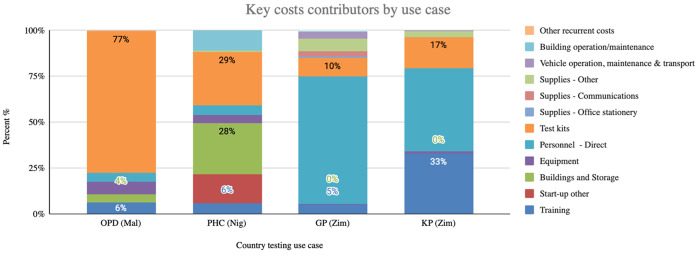
Key costs contributors by use case.

In Malawi, test kits were major cost contributors (77% of total costs); in Nigeria it was test kits (29%), building and storage (28%) and other start-up costs (16%); and in Zimbabwe it was personnel (38%) and COVID-19 tests kits (10%) for GP clinics and training (33%), personnel (25%) and tests kits (17%) for KP clinics) (Fig 2).

The costs per COVID-19 test varied across the sites within the same country and use case: they ranged from $8.56 to $11.68 in Malawi, $16.23 to $18.00 in Nigeria and from $10.76 to $56.40 and $4.19 to $209.09 in Zimbabwe respectively (Table 5).

**Table 5:** Summary COVID-19 costing data cross sites.

Country	Use case	Total costs	Min	Max	Average costs	Min	Max
**Malawi**	PHC	$256,672	$6,425	$36,810	$9.73	$8.56	$11.68
**Nigeria**	PHC	$13,418	$1,269	$5,337	$13.99	$16.23	$18.00
**Zimbabwe**	NGO GP	$41,765	$1,974	$15,064	$19.95	$10.76	$56.40
**Zimbabwe**	NGO KP	$7,054	$234	$1,406	$10.11	$4.19	$209.09

NGO – Nongovernmental organisation; KP - Key population; GP – General population.

**Fig 3:**
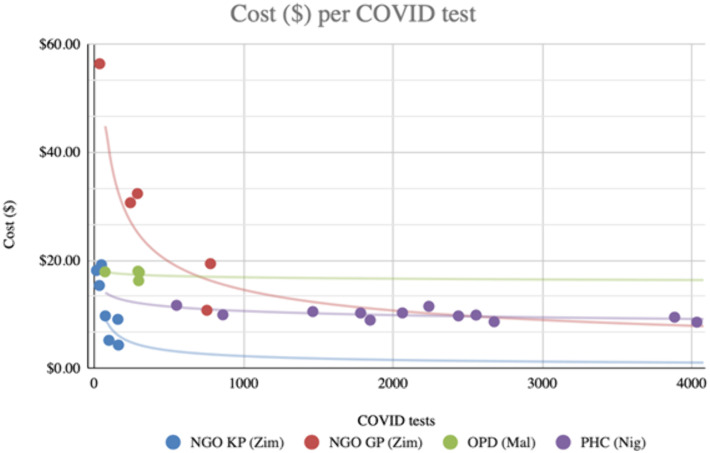
Costs per COVID-19 test by site and quantity in 2025 US$.

Fig 3 shows these unit costs plotted against the scale of COVID-19 tests across testing sites for the three countries (Fig 3). Scale economies are evidenced for some use cases by the trend towards lower unit costs for higher test throughput sites as fixed costs are spread across variable numbers of tests.

### Sensitivity analysis

**Fig 4:**
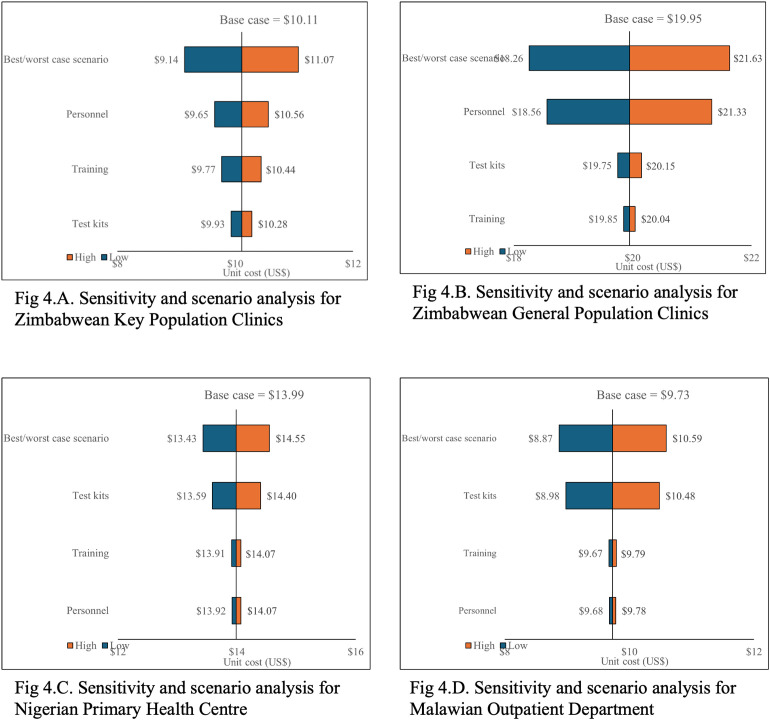
Sensitivity and scenario analysis across countries.

In deterministic sensitivity analysis (univariate and scenario analyses) we varied key input parameters including training, personnel and number of tests conducted (or test kits distributed) -/ + 10% to reflect different programmatic scenarios such as scale-up through existing cadres and therefore no training, future increases in human resources costs and changes in uptake of testing. Sensitivity analysis results were robust to variations in input parameters. Cost results were highly sensitive to variations in number of tests for the Malawi OPD and Nigeria PHC. In the Zimbabwe GP and KP use cases results were highly sensitive to personnel costs (Fig 4).

As we were not able to collect above site (head office costs) costs in the OPD (Malawi) and PHC (Nigeria) use cases we present Table 6 below which displays the cost results when above site level costs are removed from the Zimbabwe NGO GP and KP use cases.

**Table 6:** Total programme cost summary by expenditure level (in 2023 US$).

Country	Malawi		Nigeria		Zimbabwe			
Use case: Total tests Duration in months: Range	OPD: 26,386 16 months: March 2022 to June 2023		PHC: 959 12 months: May 2022 to June 2023		NGO GP: 2,094 12 months: May 2022 to June 2023		NGO KP: 698 12 months: May 2022 to June 2023	
Cost categories $ (by input)								
Above site level costs	**Cost US$**	**%**	**Cost US$**	**%**	**Cost US$**	**%**	**Cost US$**	**%**
Total capital costs (above site)					**$ 60**		**$ -**	**–**
Total recurrentcosts (above site)					**$ 19,289**		**$ 219**	
Total above site level costs					**$ 19,349**	**46%**	**$ 219**	**3%**
Site level costs								
Total capital costs	**$44,617**		**$7,226.57**		**$ 2,245**		**$ 2,400**	
Total recurrent costs	**$212,054.63**		**$6,191.57**		**$ 20,171**		**$ 4,435**	
Total site level costs	**$256,671.63**	**100%**	**$13,418.14**	**100%**	**$ 22,416**	**54%**	**$ 6,835**	**97%**
Total programme costs	**$256,671.63**	**100%**	**$13,418.14**	**100%**	**$ 41,765**	**100%**	**$ 7,054**	**100%**
Costing outcomes								
# COVID-19 tests	**26,386**		**959**		**2094**		**698**	
Cost per COVID-19 test (all levels)					**$19.95**		**$10.11**	
Cost per COVID-19 test (site level only)	**$9.73**		**$13.99**		**$10.70**		**$9.79**	
# COVID-19 + tests (assuming 5.53% positivity)	**493**		**12**		**67**		**39**	
Cost per COVID-19 + test (all levels)					**$623.28**		**$182.64**	
Cost per COVID-19 + test (site level only)	**$520.63**		**$1,118**		**$334.53**		**$176.96**	

NGO – Nongovernmental organisation; KP - Key population; GP – General population.

In the KP use case in Zimbabwe 97% ($ 6,835) of total programme costs were incurred at site level whereas 54% ($ 22,416) of costs in the GP use case were above site level. Costs per COVID-19 test across the country use cases, while excluding the above site level costs (Table 6) in Zimbabwe, were $9.73 for Malawi OPD, $13.99 for the Nigeria PHC and $10.70 and $9.79 in the Zimbabwe GP and KP uses cases.

## Discussion

In this paper we report the findings of a cross-country economic cost evaluation of COVID-19 testing. It is to the best of our knowledge the first published study focusing on the provider costs of COVID-19 testing across Malawi, Nigeria and Zimbabwe. Although COVID-19 no longer constitutes a public health emergency of international concern, the results of this cost analysis still carry relevant implications for testing in LMICs and for pandemic preparedness in general, particularly as WHO continues to consider COVID-19 an established and ongoing health issue requiring long-term, sustained management [1–4]. We were able to establish important findings despite varying country contexts and design characteristics of testing use cases. From a health provider perspective, the mean costs per SARS-CoV-2 testing across our three countries ranged from: $9.73 in the Malawi OPD (lowest) and $13.99 in Nigeria PHC to $10.11 and $19.95 in the Zimbabwe KP and GP models respectively.

Variations in costs across the country contexts and use cases may emanate from the differences in use case characteristics specifically PHC in Malawi and Nigeria (government run) versus NGO models (KP and GP) in Zimbabwe. NGO models generally tend to be more expensive to run including employment of higher level cadres and higher salaried staff. Differences are also evident in the design characteristics of the KP and GP use cases within Zimbabwe which see the former embedded in public health facilities located in rural or smaller urban sites with lower (or none) rentals and other running costs. The GP clinics on the other hand are located in high throughput urban zones which attract high rentals and other running costs. The higher costs associated with the NGO models may however be necessary as they help target and reach specific high risk populations such as sex workers more effectively.

Only few studies evaluating the costs of SARS-CoV-2 RDT testing within LMIC settings have been published prior to our analysis. A micro-costing study in five countries (Brazil, Georgia, Malaysia, Ethiopia and the Philippines), with most implementation modalities being workplace distribution models, found high variation with costs per test ranging from $2.44 to $12.78 [45]. The upper boundary of costs is similar to our results, while the lowest costs assumed higher demand and lower test kit prices than in our contexts, which contributes in explaining the difference. It is important to note that as a self-testing model, this required less health care worker time contributing to its lower costs compared to this study. Meanwhile, a study in Mozambique [46] found a unit cost of $8.9 to $13 and another study in Brazil found costs between $14 and $18, both results being quite similar to our estimates.

In our cost analysis, costs per SARS-CoV-2 test appear variable across the three countries, between use cases and between sites. In particular, we find that unit costs fall as the number of tests conducted increases as seen in cost analyses of COVID-19 testing and other public health programmes elsewhere, suggesting scale opportunities are available. Ramping up COVID-19 tests in Nigeria where start-up costs are high may result in lesser costs as economies of scale are exploited more. In our studies of costs of testing in similar settings we have found substantial evidence of economies of scale as costs similarly displayed high variability across countries and sites and were substantially lower when distribution was at a larger scale [32,33,47]. Matsimela *et al* also observed economies of scale related to test kit distribution volume. Hansen *et al* found the cost per test distributed was initially high but decreased with programme scale-up since fixed start-up (data systems, planning and communication design) and once-off costs (training) were spread over larger numbers of tests and greater integration into routine operations ensured incrementally smaller staff costs absorbed by the provider [33].

Staffing contributes to costs substantially and if exploited more through increased testing can create substantial scale opportunities. In sensitivity analysis we found testing highly sensitive to variations in personnel costs in the Zimbabwe GP and KP use cases suggesting cost reductions strategies are implemented to more efficiently utilise human resources. Programmes for COVID-19 testing and other future pandemics can aim to optimise costs by more intensively using personnel on tasks that require their expertise and shifting the actual testing to testers themselves through self-testing. In previous analyses we have observed that staff costs tend to decline with service maturity as programme teams become more efficient through learning by doing [23]. Start-up costs and staff planning, and management costs dedicated to testing programmes have been shown to reduce during routine implementation, creating lower programme and unit costs. Sande *et al* found personnel costs were a key cost driver in HIV self-test kit distribution when integrated into public health facilities across four sub-Saharan African countries [32]. Matsimela *et al* also found personnel as one of the highest cost drivers in their analysis, with the cost ranging from $5.30 to $6.29 [33].

Finally, in our analysis, test kits prices are also a key cost contributor, further highlighting potential opportunities for cost reductions. Again in sensitivity analysis this finding persisted with costs per test in the Malawi OPD and Nigeria PHC highly sensitive to variations in test uptake and suggesting cheaper test kits could help reduce costs of testing. Test kit prices and other medical consumables have been known to contribute substantially to costs of new interventions, drugs and devices [32,33,47]. Cost reductions have then been observed when prices of these supplies are negotiated down or fall over time with increasing competition [23]. Sande *et al* and Matsimela *et al* also found test kit price was a key cost driver in their analysis of HIV self-test kit distribution integrated into public health facilities across the four sub-Saharan African countries mentioned above [32,33]. In Mozambique, Manjate et al found test kit costs varying from $2.1 to $6.2 depending on the brand [46]. Global Fund now sets the SARS-CoV-2 RDT reference price at $0.6-2.5, substantially lower than unit costs in this paper [48]. It will therefore be important, when choosing between competing testing algorithms for governments in LMICs, to take advantage of lower priced tests as test availability broadens, and for funders to influence global pharmaceutical prices further down where possible.

### Limitations

This economic cost study faced some limitations. First, the cost estimates were drawn from a relatively small sample size limiting the generalisability of our results. The economics team in some instances struggled to access financial expenditure data from in-country implementing partners limiting our ability to analysis above site costs in some country use cases. We were however able to present our costs both including and excluding the above site level costs in order to more precisely compare costs across country use cases.

In addition and despite the aforementioned challenges our study approach has some important strengths to note. Although there is always potential upward bias in the observed time counsellors spend on service provision due to the Hawthorne effect (deviation from normal behaviour under observation by health providers) we believe actual time-and-motion data collection as conducted in this study rather than retrospective self-reporting by individuals (which is subject to recall bias) ensured more accurate time estimates and ultimately better allocation of personnel salaries. Although we experienced challenges in accessing expenditure data in some of the use cases our mixed methods approach allowed us to collect both site level, ensuring bottom-up accuracy at tracking and assigning resource use, and some above site expenditure data which allowed us to include overheads and thus ensure full economic costing.

## Conclusions

We estimated antigen-based COVID-19 testing by professional health-care workers to cost between $9.93 and $19.95 in Malawi, Nigeria, and Zimbabwe from a health system perspective. These cost estimates can support policymakers in deciding on the most suitable approach for scaling up COVID-19 testing programmes and for future pandemic preparedness efforts. Furthermore these conclusions help health systems identify and prioritize affordable testing methods that can be scaled effectively during a pandemic. They enable governments and organizations to allocate sufficient resources for testing and other critical interventions. The cost estimate help assess the feasibility of scaling testing efforts in various contexts, ensuring readiness for rapid response during outbreaks.The data also highlights the need to ensure that testing is accessible and affordable for all populations, particularly in low-resource settings. However, varying epidemiological and demographic settings may lead to deviations from these estimates in other LMICs.

## Supporting information

S1 File Narrative description of the COVID testing use cases across countries. (DOCX)

S2 File Definitions of cost category and cost inputs. (DOCX)

S1 Table Cost allocation factors across the interventions by cost input type. (DOCX)

S2 Table Assumptions guiding costing. (DOCX)

S1 Checklist Inclusivity questionnaire. (DOC)
