# Supplementary material for: Provider costs of professional COVID-19 rapid antigen testing in low-income settings
Source: PLOS Glob Public Health. 2025 Oct 8;5(10):e0005251. doi: 10.1371/journal.pgph.0005251 (PMC12507259; doi:10.1371/journal.pgph.0005251)
Supplement: S1 File — (DOCX) [file pgph.0005251.s001.docx]

**S1 File: Narrative description of the COVID test use cases**

**Out-patients department (Malawi)**

In Malawi testing was done at 12 per-urban health care facility outpatient departments (OPD) serving the general population. Facility level provider-administered test involved testing of COVID-19 by health care workers. Secondary distribution self-testing involved providing self-test kits, and instructions/demonstration on how to perform own COVID-19 testing, to an index case to distribute to contacts who did their own testing. All participants were aged 16 years or older.

**Routine provider-delivered diagnostic CV19 Ag-RDT testing and self-testing in symptomatic out-patients**

Symptomatic individuals accessing OPD

Refusal – exit

Propose COVID self-testing or provider-led testing

Provider-led testing

Negative – exit

Consent for NAAT confirmation and sequencing if not part of recognized outbreak

Positive – Receive KN95 masks & health education

If severe or vulnerable, refer for safer management

If selected and consenting, contact tracing

**Primary health Centre (Nigeria)**

In Nigeria Society for Family Health (SFH) worked on introduction, and early implementation scale up of routine diagnostic CV19 Ag-RDT through primary care and community facility-based provider testing. Community facility-based provider testing was rolled out through patent and proprietary medicine vendor stores (PPMV) selected based on being in locations underserved by existing primary health clinical provision. PPMVs which are drug stores serving a generally poorer socioeconomic demographic, are often the primary space people present when they are unwell.

**Primary care facility-based provider testing pathway**

All clinic attendees and staff meeting national COVID-19 testing criteria offered provider-led testing

Refusal – exit study

Provider-delivered Ag-RDT for all meeting criteria by SFH and accepting testing

Positive – Consent to provide confirmation with PCR + high quality masks

Negative – counsel on possibility of being positive + referral to local clinical service

Managed in line with National guidelines

**Integrated NGO use case (Zimbabwe)**

Population Solutions for Health (PSH) also implemented provider-delivered testing integrated with their ongoing clinic and workplace-based programmes within their integrated New Start Centre network.

**NGO GP Patient Pathway**

Rapid COVID-19 testing for

• Walk in HIV/SRH clients at 6 New Start Centres exhibiting Covid-19 symptoms

• All staff (program, support, office, field based) during routine testing and when symptomatic

Positive

Confirm with PCR

Negative no follow-up

Positive

Contact Listing.

Contacts are offered the Self-test either at home or at the site

The Tester and the Client should agree on whether to do the self-tests at home or at the sites

Home Tests

- For the home use model, the PSH staff is to give instructions to the index on how to carry out the self-test with their contacts. The distributor will follow up on the results of the tests done in the time agreed with the index.

Site Testing

- If the contacts are to visit the site for the tests, they will be offered the provider assisted Self tests.

Low Risk and mild symptoms

Isolate/quarantine

Risk Profiling and symptom screening using standard checklist

Medium/high Risk and nor/moderate symptoms

Isolate/Quarantine

Severe

Follow the MoHCC protocol

➢ Isolate the client

➢ Contact the Rapid Response Team & refer the client for further assistance

10% given confirmatory PCR

Negative no follow-up

**Key Population use case (Zimbabwe)**

At KP clinics run by CeSHHAR Zimbabwe, sex workers presenting to 11 static Sisters program clinics underwent screening for COVID-19 symptoms as they arrived at the clinic according to current standard of care and those who had symptoms or reported being contacts of individuals who had tested positive for COVID-19 were offered provider-delivered testing using antigen tests.

*Fig A: Outline of COVID-19 testing strategy among KPs in Zimbabwe*

**Universal testing in the national Key Population (32) program**

Rapid COVID-19 testing of SW attendees at all clinics

**Positive**

**Confirm with PCR**

**Negative**

**No follow-up**

**Negative**

**Positive**

**Soon after positive: Symptom severity screening using standard checklist**

**Mild/no symptoms and mild risk**

- **Isolate/Quarantine**
- **Give food-pack**
- **If symptoms worsen to medium/severe adopt respective protocols**

**Severe symptoms:**

**Refer for hospitalisation**

**Moderate symptoms and/or moderate/high risk**

- **Isolate/Quarantine**
- **Given pulse oximeter**
- **1 visit & 1 phone call – complete symptom checklist**
  - **If severe refer to hospital**
  - **If mild/medium continue follow-up**
- **Give food-pack**

10% given confirmatory PCR

**No follow-up**
