# Supplementary material for: Provider costs of professional COVID-19 rapid antigen testing in low-income settings
Source: PLOS Glob Public Health. 2025 Oct 8;5(10):e0005251. doi: 10.1371/journal.pgph.0005251 (PMC12507259; doi:10.1371/journal.pgph.0005251)
Supplement: S2 File — (DOCX) [file pgph.0005251.s002.docx]

**S2 File: Definitions of cost category and cost inputs**

**Start-up costs**

Start-up costs, including the costs incurred in providing training and sensitization activities, and all costs incurred during the period of intervention design and preparation were treated as a capital cost as benefits of such investments would be expected to accrue to programmes over longer periods.

**Capital costs,** include building space, equipment and vehicles.

**Recurrent costs**

Recurrent costs included the cost of personnel (management and supervision and program staff), and project operational activities which included vehicle operation costs such as fuel, insurance and maintenance for vehicles, building operations and maintenance, recurrent training, waste management costs and utilities. Building space included program office space, warehouses and storage spaces at health facilities within intervention districts. Building operation and maintenance costs included rentals, utilities such as electricity and water, building insurance and security. Other supplies included office stationery such as bond paper, printer cartridges, first aid kits, envelopes, maps and pens, cellphone credit/airtime and internet data as well as utensils and office snacks and teas. Other recurrent costs included indirect expenses such as consultancies, office repairs and office fuel expenses.
