# Supplementary material for: Provider costs of professional COVID-19 rapid antigen testing in low-income settings
Source: PLOS Glob Public Health. 2025 Oct 8;5(10):e0005251. doi: 10.1371/journal.pgph.0005251 (PMC12507259; doi:10.1371/journal.pgph.0005251)
Supplement: S1 Table — (DOCX) [file pgph.0005251.s003.docx]

**S1 Table: Cost allocation factors per cost input type**

| **Cost input type** | **Allocation factors to demand creation model** |
| --- | --- |
| **Start-up Costs** and c**apital Costs**  Use case development costs  Initial training costs:  Equipment costs: *Country HQ, regional & district staff*  **Recurrent Costs**  Personnel costs*: Country HQ, regional & district staff* | % of tester's trained per use case and per site  % of tester's trained per use case and per site |
| Vehicle operation & maintenance costs: *Car hire, fuel, tubes, tires* | % of client’s tested |
| Communication & Education  HIV self-test kits  Promotional & other supplies  Program related   - training - meetings   Other recurrent   - *Stationary* - *sim-cards & airtime* | % of client’s tested  % IEC material allocated  % active tester's  % of client’s tested |
