# Supplementary material for: Provider costs of professional COVID-19 rapid antigen testing in low-income settings
Source: PLOS Glob Public Health. 2025 Oct 8;5(10):e0005251. doi: 10.1371/journal.pgph.0005251 (PMC12507259; doi:10.1371/journal.pgph.0005251)
Supplement: S2 Table — (DOCX) [file pgph.0005251.s004.docx]

**S2 Table: Assumptions guiding costing**

| **Assumption/Decision** | **Value** |
| --- | --- |
| Discount rate | *3%* |
| **Exchange rate** |  |
| *Malawi* | *1,034.870 (USD/MWK)* |
| *Nigeria* | 460.702 (USD/NGN) |
| *Zimbabwe* | *USD1 = USD1* |
| **COVID kit price** |  |
| *Malawi* | $4.1 |
| *Nigeria* | $7.5 |
| *Zimbabwe* | $2.0 |
| **Lifespan (economic life) of capital inputs** |  |
| *Use case development costs* | *5* |
| *Start-up costs* | *5* |
| *Initial training* | *4* |
| *Buildings* | *35* |
| *Vehicles* | *10* |
| *Furniture/equipment* | *5* |
